# Beta Diversity of Tenebrionid Beetles (Coleoptera, Tenebrionidae) in Ningxia Grasslands and Their Driving Factors

**DOI:** 10.3390/biology14060680

**Published:** 2025-06-11

**Authors:** Changyu Xiong, Ziyu Cao, Haixiang Zhang, Ying Wang, Wei Sun, Chun Shi, Yifan Cui, Rong Zhang, Shuhua Wei

**Affiliations:** 1 College of Biological Science & Engineering, North Minzu University, Yinchuan 750002, China; 15950550587@163.com (C.X.); czy200218@163.com (Z.C.); sc450776071@163.com (C.S.); 2 Institute of Plant Protection, Ningxia Academy of Agriculture and Forestry Sciences, Yinchuan 750002, China; haixiangzhang@cau.edu.cn (H.Z.); wangying108@163.com (Y.W.); swlymyy@163.com (W.S.); sy20233243683@cau.edu.cn (Y.C.); yczhrnx@163.com (R.Z.); 3 College of Grassland Science and Technology, China Agricultural University, Beijing 100193, China

**Keywords:** Ningxia grasslands, Tenebrionidae, beta diversity, environmental factors, driving mechanisms

## Abstract

This study explored the beta diversity patterns of tenebrionid beetles and their driving factors across four grassland types in Ningxia. A total of 32 monitoring sites were surveyed, capturing 5328 individuals from 47 tenebrionid species. Permutation multivariate dispersion (PERMDISP) and non-metric multidimensional scaling (NMDS) revealed significant differences in community composition among the four grassland types (Stress = 0.091). Beta diversity decomposition indicated that species replacement primarily drives beta diversity among four temperate grassland types. Canonical Correspondence Analysis (CCA) showed that mean temperature, field water holding capacity, total soil phosphorus, biomass of Asteraceae, biomass of Leguminosae, and frequency of Leguminosae significantly explained variations in dominant species abundance (*p* < 0.05). Mantel tests revealed that beta diversity was positively correlated with differences in mean temperature, field water holding capacity, total soil phosphorus, and Asteraceae biomass (*p* < 0.01), but negatively correlated with Leguminosae frequency (*p* < 0.05). Overall, daily mean temperature was the primary driver of diversity, with synergistic effects from climate, elevation, soil, and vegetation. This study provides a comprehensive analysis of environmental factors influencing tenebrionid diversity, offering insights for grassland biodiversity conservation.

## Simple Summary

Beta diversity of insects plays a crucial role in grassland biodiversity conservation, with extensive research exploring insect diversity across different habitats. This study investigated the diversity patterns and driving mechanisms of Tenebrionidae communities in Ningxia grasslands. By analyzing community composition differences and partitioning beta diversity into replacement and nestedness components, we further identified the environmental factors influencing dominant species and the drivers shaping beta diversity distribution patterns. Our findings revealed that synergistic effects of multiple environmental factors govern their spatial distribution.

## 1. Introduction

The Ningxia Hui Autonomous Region is situated in the temperate climate zone of central eastern Asia, featuring a long and narrow territory with complex geographical variations. It covers a total area of 67,000 km^2^, with elevations ranging from 1090 to 3350 m and annual precipitation between 200 and 650 mm. The gradient effects of multiple environmental factors induced by altitude result in distinct vertical zonation of vegetation distribution, while precipitation affects the distribution pattern of species richness in Ningxia [1,2]. The northern and northwestern regions of Ningxia are situated on the southwestern margin of the Alxa Plateau, whereas its southern and southwestern sections adjoin the Loess Plateau in Gansu Province. Additionally, the northeastern and southeastern portions of Ningxia border the Ordos Plateau in Inner Mongolia. Its unique geographical location not only features a high degree of insect diversity, making it a key area for insect research, but also is the important part of the “Loess Plateau-Sichuan-Yunnan” ecological barrier and the northern sand control belt in China’s “Two-screen and three-belt” ecological security pattern, which ensures the ecological security of the upper and middle reaches of the Yellow River and North and Northwest China [3]. Although Ningxia is relatively small in size, it exhibits diverse landscapes, including plains, plateaus, high mountains, and intermountain basins, encompassing climatic characteristics representative of China’s three major natural regions [4]. The proportion of grassland area relative to the total land area ranks fourth in the country, following only Inner Mongolia, Tibet, and Qinghai. Among these, the four primary grassland types, temperate meadow steppe, temperate steppe, temperate desert steppe, and temperate steppe desert, account for 98.01% of Ningxia’s total grassland area. These ecosystems play a crucial role in carbon sequestration, soil and water conservation, and maintaining ecological security [5,6].

Ground-dwelling beetles represent one of the most biodiverse faunal groups in desert steppe ecosystems. Key families include Scarabaeidae (dung beetles), Carabidae (ground beetles), and Tenebrionidae (darkling beetles), with Tenebrionidae being the dominant family. As coleopterans (order: Coleoptera), Tenebrionida rank among the most species-rich taxa in Ningxia’s desert grasslands. While harsh desert conditions cause sharp declines in species numbers and populations of other insect groups, Tenebrionidae beetles show relative increases. First, Tenebrionid beetles exhibit enhanced assimilation capacity, enabling them to efficiently utilize living, desiccated, and even decaying plant/animal residues for nourishment, which is a capability unparalleled among other insect taxa. Second, their self-preservation mechanisms are remarkably sophisticated. They employ subelytral cavities for water storage, heat resistance, and moisture filtration, regulate physiology through integumentary pigmentation and circadian activity rhythms, and acquire water through soil-burrowing behaviors. Third, they possess specialized anatomical adaptations for water conservation. The elytra feature distolateral false flaps that enclose abdominal spiracles, exhibit fused elytral sutures (rendering wings non-openable), experience metathoracic wing degeneration, and demonstrate tightly interlocked head—thorax–elytra connections. These adaptations collectively demonstrate parallel evolution with their arid environments [4,7,8]. The diverse life strategies of tenebrionid beetles enable them to ingeniously utilize natural and animal created refugia, including burrows, subcortical spaces, root channels, various barriers, and even fecal matter, for aggregation [9,10]. They exhibit exceptionally high species diversity in arid and semi-arid regions. Ecological assessments often apply the indicator value method (IndVal), whereby species with indicator values exceeding 70% are designated as characteristic indicator taxa for specific habitat types [11,12]. Tenebrionids serve as reliable bioindicators of vegetation degradation intensity and soil desertification severity. Numerous studies highlight the critical ecological roles and conservation significance of Tenebrionidae, rendering them vital research subjects in China’s arid and semi-arid ecosystems.

Based on different biodiversity measurement approaches, ecologist Whittaker proposed using alpha, beta, and gamma diversity to characterize species diversity at three distinct levels [12,13,14]. Among these, beta diversity can be further decomposed into two components: species replacement (or turnover) and richness difference (or nestedness) [15,16,17]. Species turnover refers to the replacement of species along spatial or environmental gradients, typically occurring between communities with high speciation rates or dispersal limitations. Nestedness represents differences in species richness caused by species gain or loss, often resulting from species thinning or other ecological processes such as human disturbance or physical barriers that lead to variations in species richness among biologically impoverished areas [18,19]. The interspecific compositional differences reflected by beta diversity provide a more comprehensive understanding of diversity change processes and species’ spatial distribution patterns. These insights can inform the design of nature reserve networks and ecological corridors, optimize conservation planning, and maximize protection effectiveness, making beta diversity particularly valuable for biodiversity conservation [20]. Therefore, systematically studying the beta diversity of tenebrionid beetle communities in Ningxia grasslands and their driving factors will help identify priority areas for grassland biodiversity conservation and develop targeted restoration strategies [12,16,20].

## 2. Materials and Methods

### 2.1. Monitoring Area

**Figure 1:**
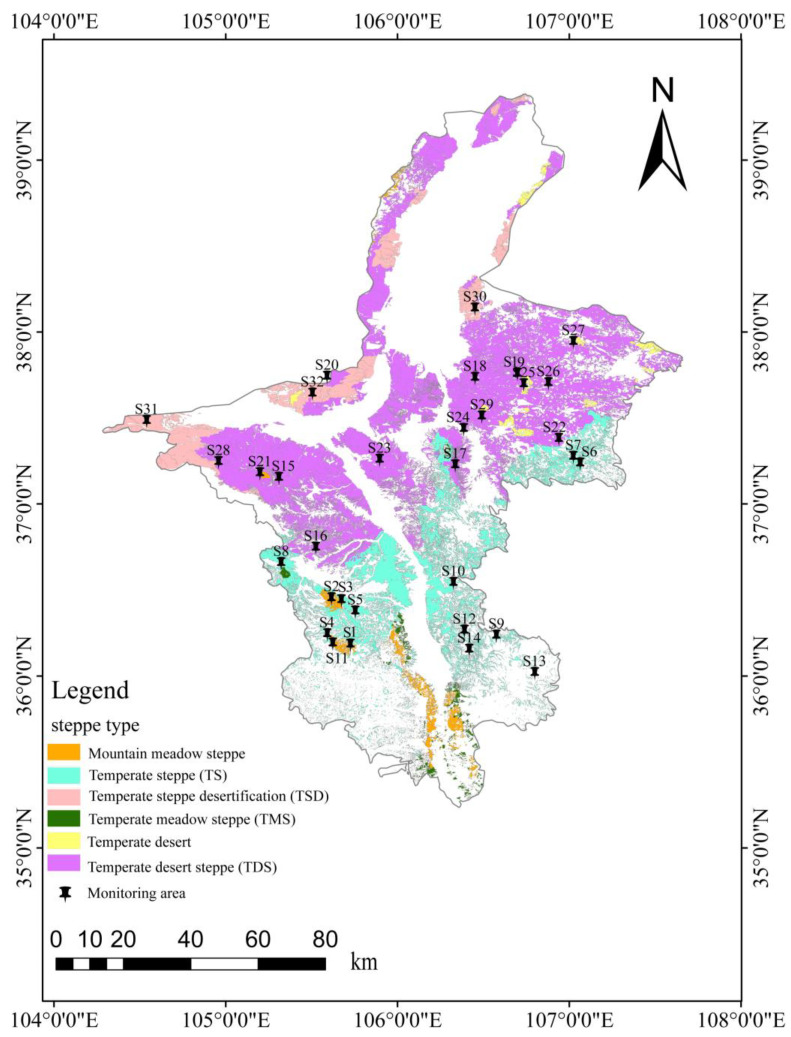
Distribution map of monitoring sites in Ningxia grasslands.

The Ningxia Hui Autonomous Region is located in the upper and middle reaches of the Yellow River in northwestern China (104°17′–107°39′ E, 35°14′–39°23′ N; Figure 1). Using GPS positioning, monitoring plots were established based on the proportional area of four grassland types. Among these, temperate desert steppe occupies the largest area in the region, with 15 plots designated. Temperate steppe, the second largest, had 11 plots. Temperate meadow steppe and temperate steppe desert, covering smaller areas, each had 3 plots. Each plot covered approximately 50 mu (≈3.33 hectares). Surveys were conducted across all four grassland types to collect tenebrionid beetles, along with corresponding climate, soil, and vegetation data for analysis.

### 2.2. Tenebrionid Beetle Collection

During the peak pest outbreak period (May, July, and September), sampling was conducted using the five-point sampling method equipped with traps. Disposable plastic cups were buried in the ground after soil removal with an auger, with the cup rim flush with the soil surface. Each trap was filled to one third of its height with trapping solution (ethylene glycol/water = 1:2 by volume, with added detergent). After 7 days, trap conditions were inspected for sample collection status and potential damage by herders, with prompt replenishment of trapping solution and replacement of compromised traps, and after 14 days, insect samples were collected and preserved in 75% ethanol [6]. The samples were transported to the laboratory for pinning and mounting. Species identification and documentation were performed using the following reference materials: *Colored pictorial handbook of grassland insects in Ningxia* [21], *Fauna of the beetles from Ningxia, China* [22], and *Insects of Helan Mountain in Ningxia* [23]. The dominant species are determined based on the proportion of the number of individuals of various insects to the total number of individuals in the community. Species with a proportion of less than 1% are rare species, those with 1% to 10% are common species, and those with more than 10% are dominant species [6,24].

### 2.3. Environmental Data Collection

#### 2.3.1. Soil Environmental Data Collection

**Figure 2:**
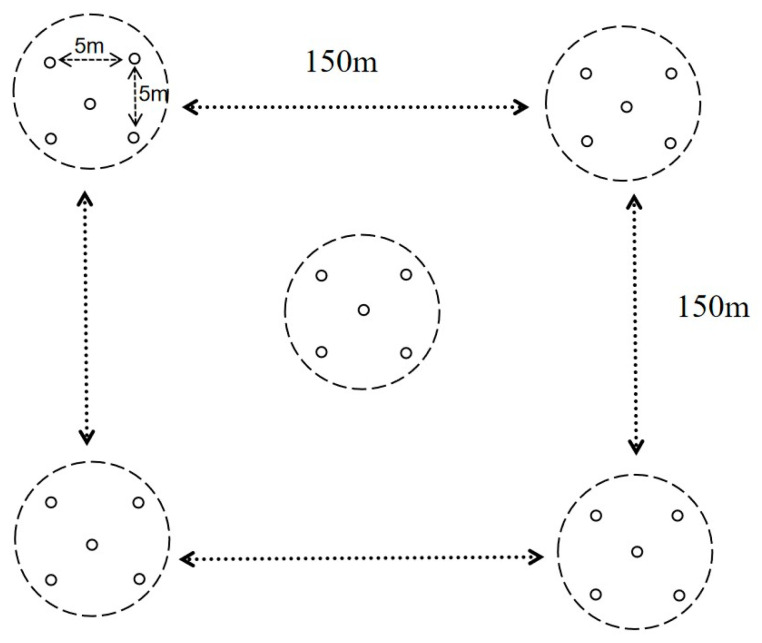
Five-point sampling diagram.

During the same period, five soil samples (0–20 cm depth) were randomly collected using the five-point sampling method around each plot where tenebrionid beetles were captured (Figure 2). These subsamples were immediately composited into one representative sample for physicochemical analysis, including organic matter, available potassium, total phosphorus, available phosphorus, soil pH, and alkali hydrolysable nitrogen; undisturbed soil cores (0–10 cm depth) were collected using soil cores for measurement of soil bulk density, field water holding capacity, saturated water holding capacity, capillary water holding capacity, capillary porosity, non-capillary porosity, and total porosity.

#### 2.3.2. Climate and Elevation Survey Methods

The climate data were provided by the Ningxia Meteorological Bureau. Statistical analysis was conducted on the meteorological information of 34 survey sample points near the national reference climate stations, basic meteorological stations, ordinary meteorological stations and regional meteorological stations during the sample capture period of the tenebrionid beetles (collected in the three months of May, July and September when the tenebrionid beetle population was highly prevalent). The collected parameters included mean temperature (°C), maximum temperature (°C), minimum temperature (°C), precipitation (mm), and elevation (m).

#### 2.3.3. Vegetation Data Collection

Vegetation sampling was conducted using the random sampling methodology, with a minimum distance of 50 m between quadrats. Within each monitoring area, three 1 m × 1 m quadrats (10 m × 10 m for shrub communities) were randomly established. A total of 9 quadrats were established in temperate meadow steppe, 33 in temperate steppe, 45 in temperate desert steppe, and 9 in temperate steppe desert areas. The following vegetation parameters were recorded: species composition, plant height, coverage (measured by needle point method), and frequency (determined by quadrat circle method). All vegetation within each quadrat was clipped and transported to the laboratory for aboveground biomass measurement.

### 2.4. Data Statistical Analysis

Based on species abundance data, we calculated Bray–Curtis distances and Sørensen indices between sampling plots. For beta diversity analysis of tenebrionid beetle communities across the four grassland types in Ningxia, permutation multivariate dispersion (PERMDISP) and non-metric multidimensional scaling (NMDS) were performed using the Bray–Curtis distance matrix [25]. Beta diversity was partitioned into species replacement (Repl) and nestedness (Nest) components based on Sørensen indices [26,27,28]. The tenebrionid beetle community composition harbors numerous low-abundance species. The Sørensen index is less sensitive to rare species, thereby avoiding dominance by rare taxa in community dissimilarity analyses. In contrast, the Jaccard index shows high sensitivity to rare species and fails to incorporate quantitative abundance data, rendering the Sørensen index methodologically superior for such analyses. Analytical procedures included Canonical Correspondence Analysis (CCA) between dominant tenebrionid species and individual environmental factors; collinearity exclusion of combined environmental factors (VIF > 10); CCA using screened significant environmental factors; and Mantel tests between significant environmental factors and Bray–Curtis distances (Spearman correlation with 9999 permutations) [29]. All analyses were implemented in R (R Core Team, 2025) [30] using the “cca” function in the vegan package for CCA, the “mantel” function for Mantel tests in the vegan package [31]. R version 4.4.3 was used for all data analysis and graphical outputs.

## 3. Results and Analysis

### 3.1. Beta Diversity of Tenebrionid Beetle Communities Across Ningxia Grassland Types

#### 3.1.1. Compositional Differences Among Four Grassland Types

**Figure 3:**
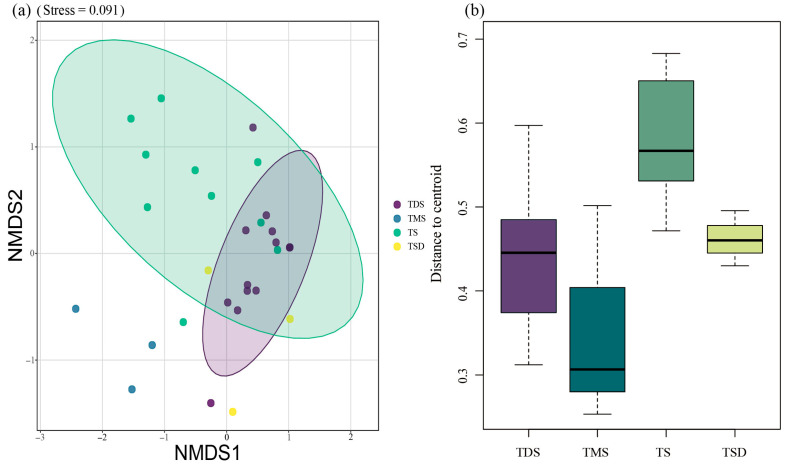
Differences in tenebrionid beetle community composition across four grassland types based on NMDS and PERMDISP analysis. Note: (**a**) Visualization of community dissimilarities, where dashed lines represent confidence ellipses for the four grassland types and dots indicate monitoring points within each grassland type. TDS is temperate desert steppe, TMS is temperate meadow steppe, TS is temperate steppe, and TSD is temperate steppe desert (the same abbreviations apply throughout the text). (**b**) The “Distance to centroid” represents the centroid distance of dissimilarity for tenebrionid beetle communities across four grassland types. The box plot illustrates the data distribution range, with the central line inside the box denoting the median (Q2), reflecting the central tendency of the data. The whiskers extend to the minimum and maximum values within the dataset.

The NMDS + PERMDISP results revealed the following: NMDS analysis showed significant between-group differences (R^2^ = 0.32, *p* = 0.001, Stress = 0.091). Based on PERMDISP results (average distance to centroid: TDS = 0.44492, TMS = 0.3539, TS = 0.5815, TSD = 0.4619, F = 2.1713, Pr (>F) = 0.003), significant differences in the beta diversity of tenebrionid beetle communities can be confidently established across the four grassland types (Figure 3).

#### 3.1.2. Partitioning of Tenebrionidae Beta Diversity Across Four Ningxia Grassland Types

**Figure 4:**
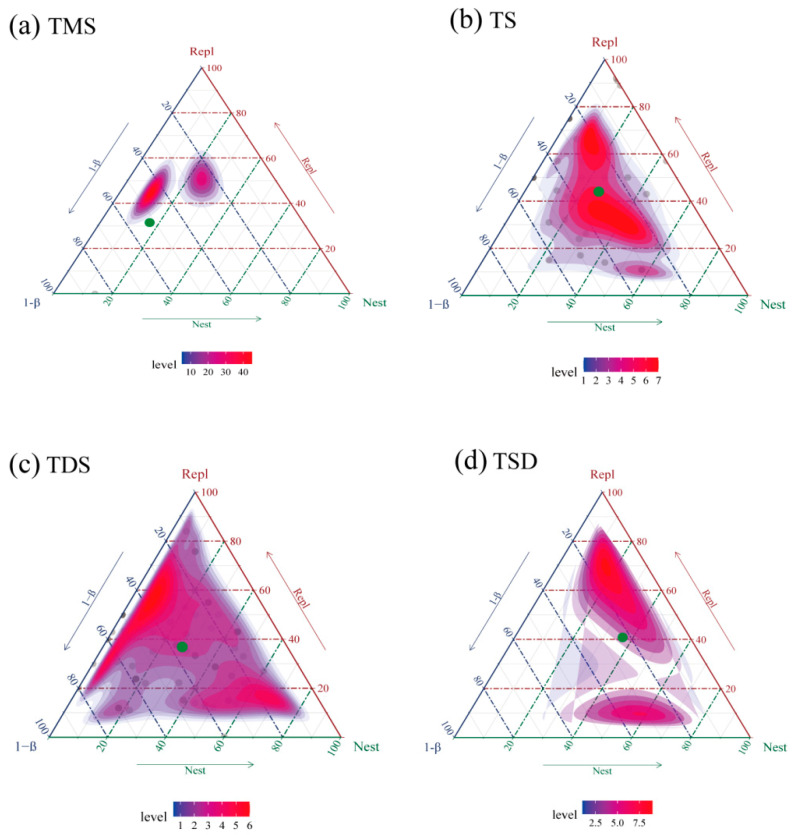
Partitioning of beta diversity in tenebrionid beetle communities across four grassland types. Note: Ternary plots visualize beta diversity and its two components for four grassland types. Green dots represent mean values for each grassland, while panels illustrate the relationship between the two partitioned components (Repl vs. Nest) for TMS, TS, TDS, and TSD, respectively. (**a**) TMS is temperate meadow steppe. (**b**) TS is temperate steppe. (**c**) TDS is temperate desert steppe. (**d**) TSD is temperate steppe desert.

The beta diversity of tenebrionid beetle communities was decomposed into species replacement (Repl) and nestedness (Nest) components using Sørensen indices. Key findings for each grassland type (illustrated in Figure 4a–d) include the following: temperate meadow steppe: total beta diversity: 0.2414; dominant process: species replacement (65.21% contribution); temperate steppe: total beta diversity: 0.3495; dominant process: species replacement (62.92% contribution); temperate desert steppe: total beta diversity: 0.3201; dominant process: species replacement (57.49% contribution); temperate steppe desert: total beta diversity: 0.3852; dominant process: species replacement (52.59% contribution) (Figure 4).

### 3.2. Environmental Factors Influencing Tenebrionid Community Diversity in Ningxia Grasslands

#### 3.2.1. Environmental Factors Affecting Dominant Tenebrionid Species

**Figure 5:**
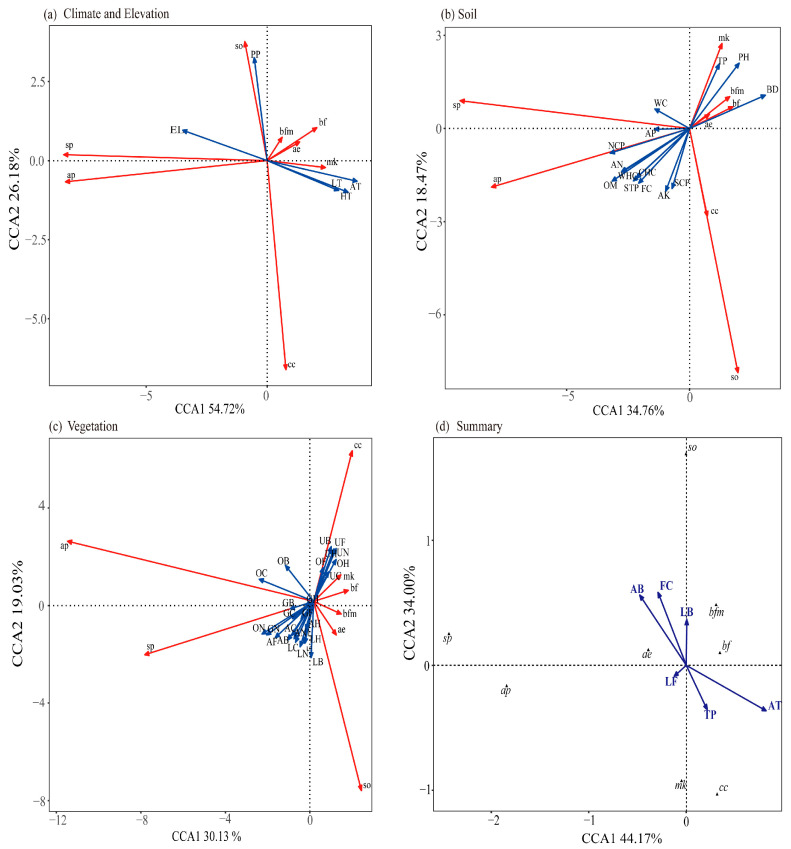
CCA ordination diagram illustrating environmental factors influencing tenebrionid beetle community composition. Notes: (**a**–**d**) Red arrows: Relative abundance of dominant tenebrionid species. Blue arrows: Environmental variables; *sp* is *Scytosoma pygmaeum*, *cc* is *Cyphogenia chinensis*, *ap* is *Anatolica potanini*, *bf* is *Blaps femoralis*, *bfm* is *B. femoralis medusula*, *ae* is *A. ebenina*, *mk* is *Microdera kraatzi*, and *so* is *Scytosoma opaca*. (**a**) Climate and Elevation: EL is elevation (m), PP is precipitation (mm), AT is mean daily temperature (°C), HT is maximum daily temperature (°C), and LT is minimum daily temperature (°C). (**b**) Soil: BD is soil bulk density, WHC is saturated water holding capacity, CHC is capillary water holding capacity, FC is field water holding capacity, NCP is non-capillary porosity, SCP is capillary porosity, STP is total porosity, OM is organic matter, PH is soil pH, AK is available potassium, TP is total soil phosphorus, AN is alkali hydrolyzable nitrogen, AP is available phosphorus, and WC is soil water content. (**c**) Vegetation: Gramineae: GC is Gramineae cover, GF is Gramineae frequency, GB is Gramineae biomass, GH is Gramineae height, GN is Gramineae species number, LC is Leguminosae cover, LF is Leguminosae frequency, LB is Leguminosae biomass, LH is Leguminosae height, and LN is Leguminosae species number. Asteraceae: AC is Asteraceae cover, AF is Asteraceae frequency, AB is Asteraceae biomass, AH is Asteraceae height, and AN1 is Asteraceae species number. UC is shrub cover, UF is shrub frequency, UB is shrub biomass, UH is shrub height, and UN is shrub species number. OC is other plant cover, OF is other plant frequency, OB is other plant biomass, OH is other plant height, and ON is other plant species number. (**d**) Filtered variables: CCA of dominant species vs. screened variables (see main text).

Canonical Correspondence Analysis (CCA) results revealed the following: Regarding climate and elevation factors, CCA1 and CCA2 collectively explained 80.90% of the variation (CCA1: 54.72%; CCA2:26.18%). Significantly affected species (*p* < 0.05) included *Scytosoma pygmaeum*, *Cyphogenia chinensis*, *Anatolica potanini* and *Blaps femoralis*. Non significant effects were noted on *B. femoralis medusula*, *A.ebenina*, *Microdera kraatzi* and *Scytosoma opaca* (Figure 5a). Soil physicochemical properties explained 53.23% of the variation (CCA1: 34.76%; CCA2: 18.47%); four dominant species—*Scytosoma pygmaeum*, *Scytosoma opaca*, *Blaps femoralis* and *Anatolica potanini*—were significantly influenced by seven soil environmental variables (*p* < 0.05). Four dominant species—*Blaps femoralis medusula*, *Anatolica ebenina*, *Microdera kraatzi* and *Cyphogenia chinensis*—showed no significant responses to soil environmental variables (Figure 5b). Additionally, vegetation factors collectively explained 49.16% of the variation in CCA, with the first and second axes (CCA1 and CCA2) accounting for 30.13% and 19.03% of the total variation, respectively. Four dominant species—*Scytosoma pygmaeum*, *Cyphogenia chinensis*, *Scytosoma opaca* and *Anatolica potanini*—were significantly influenced by eleven vegetation variables (*p* < 0.05), while vegetation factors had no significant effects on *B. femoralis medusula*, *A. ebenina*, *Blaps femoralis* and *Microdera kraatzi* (Figure 5c). After merging individual environmental variables and excluding collinearity (variance inflation factor, VIF > 10), the screened environmental variables in CCA revealed that the composition of dominant tenebrionid species was significantly influenced by mean temperature, field water holding capacity, total soil phosphorus, Asteraceae biomass, Leguminosae biomass and Leguminosae frequency (*p* < 0.05) (Figure 5d).

#### 3.2.2. Driving Factors of Tenebrionid Beetles’ Beta Diversity Distribution in Ningxia Grassland

**Figure 6:**
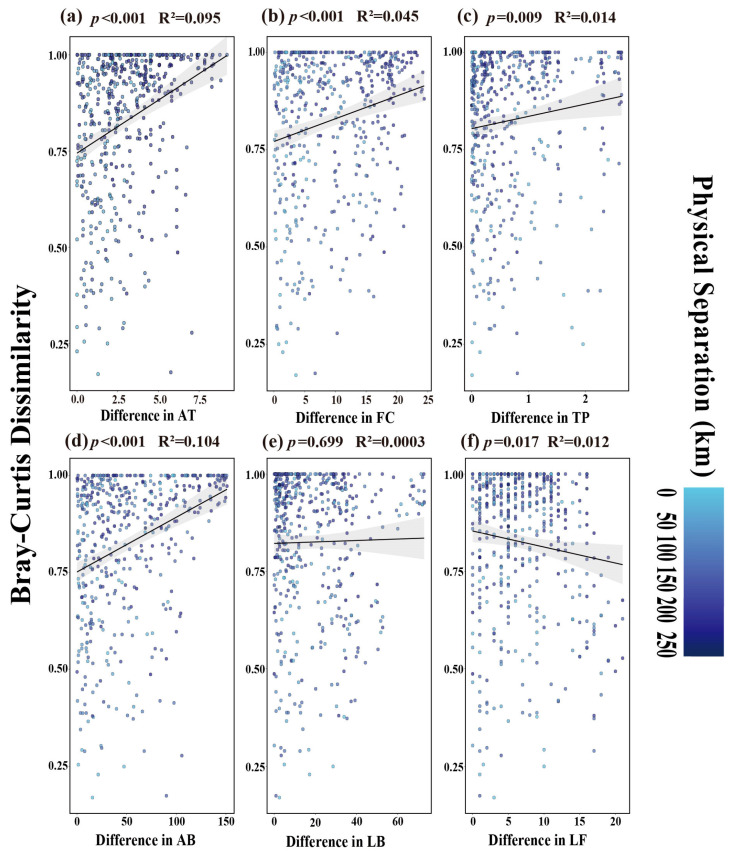
Mantel test scatterplots of Bray–Curtis distance correlations with environmental factors. Note: (**a**–**f**) display Mantel test results between tenebrionid community beta diversity. Darker scatter points indicate greater geographical distance between sample pairs. Similar colors denote spatially proximate sample pairs. (**a**) Mean temperature (AT). (**b**) Field water holding capacity (FC). (**c**) Total soil phosphorus (TP). (**d**) Asteraceae biomass (AB). (**e**) Leguminosae biomass (LB). (**f**) Leguminosae frequency (LF).

Mantel test results demonstrated that Tenebrionid beta diversity increased significantly with differences in mean temperature (R^2^ = 0.095, *p* < 0.001) (Figure 6a), differences in field water holding capacity (R^2^ = 0.045, *p* < 0.001) (Figure 6b), differences in total soil phosphorus (R^2^ = 0.014, *p* = 0.009) (Figure 6c) and differences in Asteraceae biomass (R^2^ = 0.104, *p* < 0.001) (Figure 6d). Tenebrionid beta diversity decreased significantly with differences in Leguminosae frequency (R^2^ = 0.012, *p* = 0.017) (Figure 6f). No significant correlation with differences in Leguminosae biomass was observed (R^2^ = 0.0003, *p* = 0.699) (Figure 6e).

## 4. Discussion

### 4.1. Beta Diversity of Tenebrionid Beetle Communities Across Ningxia Grassland Types

#### 4.1.1. Community Composition Differences Among Four Grassland Types

Tenebrionid beetles occupy unique ecological niches as crucial components of food webs and decomposers of fungi and decaying wood. Saprophagous tenebrionid beetles, such as species from the Tentyriini, accelerate dung decomposition by consuming livestock feces, particularly sheep and yak excrement, in grassland ecosystems. They actively participate in energy flow and material cycling within grassland ecosystems, playing indispensable functional roles in arid and semi-arid regions. Insects occupying similar ecological niches may exhibit analogous patterns in resource utilization and environmental adaptation, particularly in terms of food resource exploitation and habitat selection [29,32]. The NMDS analysis elucidated both divergences and convergences in community composition among grassland types, providing critical insights into species interactions and ecological processes within these ecosystems (Figure 3).

#### 4.1.2. Partitioning of Beta Diversity in Tenebrionid Communities Across Four Grassland Types

Beta diversity partitioning elucidates mechanisms underlying species replacement and nestedness patterns, revealing how insect diversity and community composition vary across ecosystems (Figure 4). Nestedness occurs when species-poor sites form subsets of species-rich sites [19], potentially driven by habitat heterogeneity that limits species distributions, resulting in reduced richness but higher similarity among depauperate sites. This pattern is commonly observed in ecosystems characterized by intense disturbance or fragmentation, where nestedness and richness differences are typically high, while species replacement is relatively low [20]. Previous studies suggest that physical dispersal barriers, reduced niche availability across sites, selective colonization/extinction processes, or environmental filtering may explain high nestedness proportions [28].

In this study, beta diversity decomposition indicated that species replacement primarily drives beta diversity among four temperate grassland types, reflecting greater compositional dissimilarity driven by distinct species assemblages rather than richness disparities [17,19]. Environmental gradients (e.g., temperature, soil properties) underpin turnover-dominated systems, where species reach their ecological limits and are replaced along climatic/environmental axes [28]. This finding aligns with global patterns in Tenebrionidae beta diversity [20]. By partitioning beta diversity components, we quantified the relative contributions of species replacement vs. nestedness to community divergence. Integrating climate, edaphic and species-specific factors further clarified how biotic and abiotic drivers collectively shape arid-land beetle biogeography [17].

### 4.2. Driving Factors of Beta Diversity in Ningxia Grassland Tenebrionid Communities

#### 4.2.1. Environmental Factors Influencing Dominant Tenebrionid Species

Higher temperatures promote speciation and provide favorable conditions for species evolution, aligning with our findings [7,33,34]. However, some tenebrionid species exhibited negative responses to mean daily temperature; studies have shown that ground-dwelling tenebrionid diversity declines sharply with increasing elevation [35], consistent with our results. Compared to arboreal species, terrestrial tenebrionids are more sensitive to environmental changes and exhibit stronger average adaptive capacities to harsh conditions than interspecific competition [34].

Globally, heterogeneity in vegetation, soil types and climate has been demonstrated to shape the abundance and composition of darkling beetle communities in arid and semi-arid ecosystems [9]. The habitat heterogeneity hypothesis posits that regions with greater topographic and environmental diversity support higher species richness [33]. In this study, Canonical Correspondence Analysis (CCA) revealed that climate and elevation factors exerted the strongest influence on explaining community environment relationships, followed by soil properties, with vegetation factors contributing least. We acknowledge, however, that this result may also be partly influenced by the inherently low vegetation diversity in our study region, which is characterized by a simple community structure dominated by a few drought-tolerant species (mainly Poaceae, Fabaceae, and Asteraceae). As such, the limited plant diversity may reduce the potential for vegetation to structure arthropod communities. Among energy-related variables, mean temperature exhibited the highest explanatory power (Figure 5).

Different tenebrionid species exhibited variable sensitivities to environmental drivers, suggesting differential responses to environmental factor combinations. This variability may arise from interspecific differences in interaction networks and dispersal strategies [29]. Understanding these disparities is critical for elucidating biodiversity response patterns [4].

#### 4.2.2. Drivers of Beta Diversity Distribution Patterns in Ningxia Grassland Tenebrionid Communities

Studies have shown that tenebrionid larvae feed on decomposing legume seeds or vertebrate feces [36,37,38], while extracts from Asteraceae plants exhibit insecticidal or repellent effects on tenebrionids [39,40,41], potentially explaining why Asteraceae biomass emerged as a significant driver of β-diversity differences in our results. This allelochemical-mediated mechanism was not explored in depth within the current study, but we will prioritize investigating this causal pathway in subsequent research. These differences in vegetation composition across grassland types likely contribute to beta diversity variation. The mechanistic responses of tenebrionid diversity to distinct vegetation types require further investigation.

Using Canonical Correspondence Analysis (CCA) to screen key environmental factors, subsequent Mantel tests revealed significant positive correlations between Bray–Curtis distance and differences in mean temperature (*p* < 0.001), field water holding capacity (FC) (*p* < 0.001), total soil phosphorus (TP) (*p* < 0.05) and Asteraceae biomass (*p* < 0.001). In contrast, Leguminosae frequency showed a significant negative correlation (*p* < 0.05). Prior research supports these findings: higher temperatures correlate with elevated species diversity; soil moisture deficits (critical for larval development) may constrain tenebrionid populations [8]. Soil TP is identified as a key driver of tenebrionid distribution [42]. Soil structure (e.g., pore size distribution) is emphasized as a critical environmental filter [10]. In this study, field water holding capacity (FC) and total soil phosphorus (TP) were prioritized as primary drivers of beta diversity due to their strong explanatory power. Other soil factors were excluded due to collinearity with FC/TP. Specifically, FC influences soil water retention and availability, thereby affecting larval survival—a mechanism consistent with our results (Figure 6).

## 5. Conclusions

Significant differences in beta diversity of tenebrionid beetle communities can be confidently established across the four grassland types. Regarding beta diversity, species turnover drives compositional differences among tenebrionid communities across the four grassland types.

In Ningxia grasslands, the dominant environmental drivers influencing tenebrionid diversity vary among communities. The individual environmental factors identified through screening are insufficient to solely drive beta diversity differences. They represent key drivers with high explanatory power, but the overall beta diversity variations result from synergistic interactions among multiple environmental factors. The synergistic effects of environmental factors (climate, elevation, soil, and vegetation) collectively explain the beta diversity patterns, with temperature exhibiting significantly higher explanatory power. Coordinating these factors is critical for effective biodiversity conservation. Current understanding of interactions among environmental factors in biodiversity conservation remains limited. Future research should strengthen quantitative exploration of the impact of environmental factors on biodiversity and their interactions, thereby enhancing our understanding of how different environmental variables collectively affect biodiversity. These could inform the development of an integrated “Dynamic Boundary Management—Targeted Soil Amendment—Vegetation Patch Regulation” strategy. Such a tripartite approach aims to enhance biodiversity while maintaining forage productivity, thereby establishing a quantifiable co-management paradigm for arid pastoral livestock systems.

## Data Availability

The data presented in this study are available upon request from the corresponding author.

## Abbreviations

The following abbreviations are used in this manuscript:

TDS	Temperate desert steppe
TMS	Temperate meadow steppe
TS	Temperate steppe
TSD	Temperate steppe desert
EL	Elevation (m)
PP	Precipitation (mm)
AT	Mean daily temperature (°C)
HT	Maximum daily temperature (°C)
LT	Minimum daily temperature (°C)
BD	Soil bulk density
WHC	Saturated water holding capacity
CHC	Capillary water holding capacity
FC	Field water holding capacity
NCP	Non-capillary porosity
SCP	Capillary porosity
STP	Total porosity
OM	Organic matter
PH	Soil ph
AK	Available potassium
TP	Total phosphorus
AN	Alkali hydrolyzable nitrogen
AP	Available phosphorus
WC	Soil water content
GC	Gramineae cover
GF	Gramineae frequency
GB	Gramineae biomass
GH	Gramineae height
GN	Gramineae species number
LC	Leguminosae cover
LF	Leguminosae frequency
LB	Leguminosae biomass
LH	Leguminosae height
LN	Leguminosae species number
AC	Asteraceae cover
AF	Asteraceae frequency
AB	Asteraceae biomass
AH	Asteraceae height
AN1	Asteraceae species number
UC	Shrub cover
UF	Shrub frequency
UB	Shrub biomass
UH	Shrub height
UN	Shrub species number
OC	Other plant cover
OF	Other plant frequency
OB	Other plant biomass
OH	Other plant height
ON	Other plant species number
sp	*Scytosoma pygmaeum*
cc	*Cyphogenia chinensis*
ap	*Anatolica potanini*
bf	*Blaps femoralis*
bfm	*B. femoralis medusula*
ae	*A. ebenina*
mk	*Microdera kraatzi*
so	*Scytosoma opaca*
